# Impact of short feed restriction to compensatory growth on the performance, body measurements, and carcass and non-carcass traits of Bunaji and Sokoto Gudali Nigerian bulls

**DOI:** 10.1007/s11250-026-04974-2

**Published:** 2026-03-14

**Authors:** Immanuel Ishaku Madziga, Adeola Abisoye Adesote, Bodemi Benson Jaiyeoba, José Morais Perreira Filho, Kevily Henrique de Oliveira Soares de Lucena, Leilson Rocha Bezerra

**Affiliations:** 1 National Animal Production Research Institute, Ahmadu Bello University, Sokoto Road, Zaria, Kaduna, 810105 Nigeria; 2 Department of Animal Science, Federal University of Campina Grande, Patos, Paraíba, 58708110 Brazil; 3 Odeda Farm Institute, Odeda, Ogun State Nigeria; 4 National Biotechnology Research and Development Agency, Islanku, Kogi State Nigeria

**Keywords:** Carcass, Compensatory growth, Feed restriction, Nigerian breeds, Ultrasound

## Abstract

This study investigated compensatory growth (CG) responses in Sokoto Gudali (SG) and Bunaji (BN) bulls. Forty animals (20 SG and 20 BN) were randomly assigned to two feeding levels (high-energy vs. restricted) in a 2 × 2 factorial design, with initial mean weights of 255 ± 30.0 kg (SG) and 249 ± 48.6 kg (BN). During a 30-day feed restriction phase, one group (10 SG and 10 BN) received a high-energy diet with concentrate at 2% of body weight (BW), while the second group (10 SG and 10 BN) received 1% BW. All bulls were subsequently refed a high-energy diet (2% BW) for 120 days. During restriction, bulls fed 1% BW showed reduced dry matter intake (DMI), poorer feed conversion, and decreased muscle and fat depth in the *longissimus thoracis* compared with those fed 2% BW (*P* < 0.05). During refeeding, DMI did not differ between treatments (*P* > 0.05). However, previously restricted bulls exhibited a higher average daily gain (ADG; *P* < 0.05), indicating compensatory growth. Across the entire period, bulls fed 2% BW had higher DMI but better total FCR. At the end of the refeeding period, no differences (*P* > 0.05) between supplementation level groups (1 and 2% BW) were observed for linear body measurements, carcass conformation, and fat classification. Sokoto Gudali cattle had greater subcutaneous fat, rib fat, hide weight, and larger linear carcass dimensions but lower dressing percentage and carcass conformation scores compared to BN cattle (*P* < 0.05). Bulls on the 1% BW diet achieved a mean CG index of 0.45, with no difference between breeds. Significantly, the longissimus thoracis et lumborum muscle fully recovered following refeeding. Both Bunaji and Sokoto Gudali bulls displayed clear compensatory growth after moderate nutritional restriction.

## Introduction

The greatest challenge in animal production and especially beef cattle is the availability of feed because about 75% of all variable expenses are related to feed (Kenny et al. 2018; Terry et al. 2020). Therefore, the beef industry is particularly interested in cost-cutting measures that do not sacrifice total animal performance. The potential of an animal to experience faster growth and increased efficiency upon refeeding after a previous period of restricted feeding is known as compensatory growth (CG) (Keady et al. 2021). In order to lower feed input costs, this naturally occurring rapid growth phenomena has been widely integrated into animal production systems, especially beef systems, even though it has developed naturally across many species (Ashfield et al. 2014).

According to Ashfield et al. (2014) and Fitzsimons et al. (2017), this is particularly effective in pasture-based production systems because it permits the redistribution of feed supply from periods of high cost and scarcity (such as winter) to periods of lower cost and/or abundance (such as summer pasture) while still achieving production targets. Studies show that CG can lead to significant feed cost reductions (Akintan et al. 2024). Even though CG is used all over the world, its effective application depends on number of variables that could skew the CG response. The biological basis for the accelerated growth response is unclear and not yet fully understood (Bezerra et al. 2013a; Deng et al. 2024).

The successful and efficient integration of CG into production systems may be impacted by the contradictory findings in literature. A successful CG response is influenced by both individual animal factors, such as animal age and developmental stage, breed, and sex, as well as management factors, such as the type of diet provided during the restriction and refeeding times and the degree or severity of dietary restriction executed (Cooke et al. 2020).

While there have been several studies on the CG response in connection to management factors (Martin et al. 2022; Clariget et al. 2024), there is a scarcity of evidence on animal-related aspects. There is particularly little evidence on the variance in CG response amongst different breed types, such as those with different maturation rates.

The Bunaji and Sokoto Gudali are two important Nigerian cattle breeds known for their adaptability and economic significance in livestock production (Blench 1999). The Bunaji, also called the White Fulani, is a hardy breed primarily used for dairy and beef production. It is characterized by a white coat with black or red pigmentation on the ears and muzzle. Bunaji cattle are highly adaptable to harsh environmental conditions, with strong resistance to heat and moderate disease tolerance (Olafadehan et al. 2013; Norezzine et al. 2019). Sokoto Gudali is a breed well-known for its muscular build and suitability for beef production with compact body, a short coat that varies in color (usually white, black, or brown), and they are valued for their high feed efficiency and good meat quality, making them a preferred choice for beef farming in Nigeria (Ehoche et al. 1989; Blench 1999). Both breeds play a crucial role in the Nigerian livestock industry, supporting smallholder farmers and contributing to food security. Their resilience to local climatic conditions and economic value makes them essential for sustainable cattle production in West Africa (Molina-Flores et al. 2020).

Compensatory growth (CG) permits a rapid development observed in cattle following a period of dietary restriction and subsequent refeeding. Ehoche et al. (1989) reported on compensatory growth in Sokoto Gudali cattle breeds. Because a period of feed restriction can affect body composition (Gonzaga Neto et al. 2011; Keogh et al. 2015a; Bezerra et al. 2013b) breeds with varying maturation rates may respond differently to dietary restriction and subsequent refeeding-induced CG. Consequently, the goal of this study was to look at how two Nigerian cattle breeds responded to different rations and how likely they were to demonstrate CG upon refeeding after a period of nutritional restriction. The primary objective of this study was to evaluate the effects of a 30-day feed restriction followed by refeeding on compensatory growth responses in Bunaji and Sokoto Gudali bulls. As secondary objectives, we examined breed-related differences in dry matter intake, feed conversion ratio, carcass characteristics, and linear and ultrasound body measurements.

## Materials and methods

### Animal, ethics statement, experimental design, diets and facilities

The Animal Care and Use Committee (Ahmadu, Bello University) read and approved the study’s method and procedures and gave the study approval number ABUCAUC/2021/021. The study was conducted at the National Animal Production Research Institute (NAPRI), Ahmadu Bello University, Zaria, Nigeria. Rainy season born males (*n* = 40) of Bunaji (BN) and Sokoto Gudali (SG) cows, sired through natural mating by BN or SG bulls, identified and selected from the Beef Research Programme herds of the Institute were used for this study. The animals were treated against internal and external parasites using ivermectin and inspected for general health. The average age at the beginning of the study was 28 months for both breeds, with initial mean weights of 255 (SD 30.0) kg and 249 (SD 48.6) kg for SG and BN groups, respectively.

Forty bulls were used in the experiment, comprising 20 Sokoto Gudali (SG) and 20 Bunaji (BN) animals. Within each breed, bulls were randomly allocated to one of two feeding treatments, resulting in a 2 × 2 factorial arrangement with breed (SG vs. BN) and feeding level (high-energy vs. restricted energy) as the main factors. Mean initial body weights were 255 ± 30.0 kg for SG and 249 ± 48.6 kg for BN bulls.

During the 30-day restriction phase, half of the animals from each breed (10 SG and 10 BN) received a high-energy ration consisting of concentrate supplementation at 2% of BW. The concentrate was formulated with maize (60%) as the primary energy source and cottonseed cake (39%) as the protein source, with mineral mix (1%) and bone meal (2.5%) added to meet mineral requirements. The remaining bulls (10 SG and 10 BN) were assigned to an energy-restricted treatment and received the same concentrate ingredients at 1% of BW. In both treatments, *Digitaria smutsii* hay was provided *ad libitum* as roughage (Table 1).

**Table 1:** Chemical composition of ingredients and experimental diet

Item (% DM)	Ingredients			Concentrate diet^1^
	*Digitaria Smutsii*	Maize	Cotton cake	
Dry Matter, % as fed	91.63	91.18	91.23	82.51
Organic Matter	83.90	86.76	89.01	81.23
Crude Protein	5.76	8.31	28.58	13.12
Ether Extract	4.05	8.03	11.05	17.15
Acid detergent fiber	43.12	43.27	42.35	35.11
Neutral detergent fiber	42.21	48.01	47.23	55.72
Ash	8.92	11.02	4.98	4.31

^1^Maize meal (38%), maize by-products (18.9%), cotton cake (39.1%), mineral mixture (1.50%) and bone meal (2.50%); Mineral mixture containing active element with: macrominerals (g/kg): calcium (Ca) 140 g, phosphorus (P) 100 g, magnesium (Mg) 15 g, sodium (Na) 120 g, sulfur (s) 10 g, potassium (k): 8 g; and microminerals (mg/kg): zinc (Zn) 3,000 mg; Copper (Cu) 800 mg, manganese (Mn) 2,000 mg, iron (Fe) 1,000 mg, iodine (I) 70 mg, cobalt (Co) 30 mg, selenium (Se) 20 mg

Following the restriction phase, all bulls entered a 120-day refeeding period, during which both groups received the same concentrate feeding regimen. Concentrate was offered at 2.5% BW initially and gradually increased to 3% BW, while hay remained available *ad libitum*. All animals were slaughtered on day 150 of the study.

Animals were individually fed in tie-up stalls during the differential feeding phase and housed in individual slatted-floor pens during both experiments. Fresh feed was offered daily, and refusals were weighed daily. Animals were weighed on two consecutive days at the beginning of the study, at the end of the differential feeding period, and before slaughter. Furthermore, bi-weekly weight were taken at 8:00 AM before offering feed to the animals. Dry matter intake (DMI) was measured by the difference between what was offered and what was left over.

### Diets and chemical composition

The concentrate diet was designed (Table 1) with maize meal (38.0%) and maize by-products (18.9%) as the primary energy sources and cottonseed cake (39.1%) as the protein source. To meet mineral requirements, mineral mixture (1.50%) and bone meal (2.50%) were included, aiming for a gross energy content of 16 MJ/kg DM and an average crude protein level of 16%, supporting an estimated daily weight gain of 200 g/day according to NRC (2007) guidelines. The animals received the concentrate at a rate of 1% of their live weight on a DM basis, while *Brachiaria decumbens* or *Digitaria smutsii* hay was provided *ad libitum*. Feed was allocated daily at 9:00 AM, with leftovers collected and weighed to calculate daily intake. Fresh, clean water was available at all times.

Chemical analysis of experimental feeds and refusals was carried out on representative samples. The samples were mixed and partially dried at 60° C in an oven for 72 h, and the dried samples were ground to pass through a 1.0 mm sieve and stored in containers at room temperature until chemical analysis. The ground samples were analyzed for dry matter (DM), ash, and nitrogen (N) following the procedure of AOAC (2015). Crude protein (CP) was determined by multiplying N by a value of 6.25. Neutral detergent fiber (NDF) and acid detergent fiber (ADF) were analyzed according to the procedure of Van Soest et al. (1991). Neutral detergent fiber present in residual ash was assayed with a heat stable amylase.

### Linear body and ultrasound measurements

Linear measurements were taken on day 0, and days 30, 75 and 150 using a flexible measuring tape. The following parameters were recorded: height at withers (HW), chest girth (CG), body length (BL) and pelvic width (PW). These measurements were expressed relative to body weight to assess growth patterns and body conformation.

The bulls were ultrasonically scanned five times during the study: at the beginning (day 0), the middle of the differential feeding period (day 15), the end of the differential feeding period (day 30), early in the refeeding period (day 75), and again before slaughter (day 150). A Dynamic Imaging ultrasound scanner (Concept MCV Veterinary Ultrasound scanner with 3.5 MHz probe) was used to measure M. longissimus thoracis or lumborum depth at the third lumbar vertebra, as well as fat depth at the third lumbar vertebra, the 13th thoracic rib, and the rump on the right side, as described by Conroy et al. (2010).

### Carcass traits and non-carcass components

The bulls were weighed both the day before and the morning of slaughter. These weights were averaged to provide the final live weight at slaughter (SW). All slaughter procedures complied with the animal welfare and slaughter regulations required in Nigeria. Cattle were handled, restrained, stunned, and slaughtered in accordance with the national guidelines for humane handling and meat inspection established by the Federal Ministry of Agriculture and Rural Development (FMARD), including the standards outlined in the *Nigerian Animal Welfare*,* Handling*,* Transport and Slaughter Guidelines* applicable to commercial abattoirs. All procedures respected the principles for humane stunning, avoidance of unnecessary stress, proper bleeding, and hygienic carcass handling as mandated by federal regulatory authorities.

After slaughter, cold carcass weight (CW; Hot CW 0.98) was recorded, and the dressing percentage was computed as a ratio of CW to SW. Carcass conformation and fat score were automatically recorded on a 15-point scale using video imaging analysis equipment (VBS2000, E + V, Oranienburg, Germany), as described by Hickey et al. (2007). Each bull’s non-carcass components were weighed separately, including the heart, lungs, gall bladder, liver, spleen, intestines (full), rumen excluding abomasum and omasum (full and empty), fore and hind feet, hide, kidneys, head, and perinephric/retroperitoneal fat. All measurements were determined in relation to SW.

Linear carcass measurements (Campion et al. 2009a) were recorded at 3 h post slaughter on the right side of each carcass. Carcass length, leg length, chest depth, maximum leg width, and leg thickness (width of leg from the medial splitting surface of the symphysis pubis) were recorded. After 24 h, the right side of each carcass was quartered between the fifth and sixth ribs into a pistola hind quarter (without the flank) and a fore quarter that included the flank as described by Keane and Allen (1998). The pistola was separated by cutting between the 10th and 11th ribs. The *M. longissimus thoracis* or *lumborum* shape at the 10th rib was traced onto translucent paper and measured using a digital planimeter (Placom KP-90 N, Sokkisha, Japan). The sixth to tenth rib joints (five-rib joints) were weighed and dissected to separate *M. longissimus thoracis et lumborum*, other muscles, muscle trim, total fat, and bone, as well as ligamentum nuchae/supraspinale. The dressing percentage was calculated according to equation:

$$\begin{gathered} Dressing{\text{ }}Percentage{\text{ }} = \hfill \\ {\text{ }}\left({Hot{\text{ }}carcass{\text{ }}weight/Slaughter{\text{ }}weight} \right)/100 \hfill \\ \end{gathered}$$

### Statistical analyses

The experiment was realized using a completely randomized design in a 2 × 2 factorial scheme (two Nigerian cattle breeds and two diets levels energy). The major effects in the statistical model were block, breed, ration (1 or 2% body weight), and their interaction. Variance analyses were carried out for the variables studied, using PROC MIXED from SAS^®^ (SAS, 2013, version 9.4), with a repeated statement, specifying animal as the subject and using the covariance structure that best fit the data according to Akaike’s Information Criterion (AIC). The model included the effects of dietary energy (1 and 2% BW), cattle breed (Sokoto Gudali-SG and Bunaji-BN):

$$Yijk\, = \mu \, + \,Hi\, + \,Bj{\text{ }} + {\text{ }}\left({HB} \right)ij\, + \,\varepsilon ijk,$$

where: *Yijk* = observed value of the characteristic; *µ* = overall average; *Hi* = effect of the ith energy level (i = 1, 2); Bj = jth cattle breed (j = 1, 2); HBij is the interaction between the ith hay energy level (H) and the jth cattle breed (B); *εijk* = random error associated with each observation. Levene’s test was used to assess the equality of variances for a variable of groups. Factorial ANOVA was used to examine interactions between multiple categorical variables and assess their influence on the dependent variable. The G-Power program in SAS was used to conduct power analyses. The effects of all possible variables included in the mathematical model; the effects of all possible interactions were tested. If any were not significant (*P* > 0.05), they were removed from the analyses. Data means were compared using the Tukey test at a 5% significance level.

## Results

### Dry matter (DM) Intake, body weight and live weight gain and feed conversion ratio (FCR)

There was no significant (*P* > 0.05) interaction effect between breed and feed restriction diet for DMI, FCR, initial live weight and live gain. Therefore, the variables for breed and hay were presented separately. DMI and FCR in all refeeding periods and in the total period they were affected by diet, however they were not affected by breed (Table 2). Regarding *D. smutsii* hay intake, during feeding restriction period (days 0 to 30), bulls fed concentrate supplementation at 1% BW presented high hay DMI (*P* < 0.0001) compared to bulls fed concentrate supplementation at 2% BW. In contrast, in the same period, total DM consumption (hay + concentrate) was higher in animals fed 2% BW diet (*P* < 0.0001). In the early refeeding period (days 30 to 75) and final period to slaughter (days 75 to 134), there was no effect of diet on DMI (*P* > 0.05).

**Table 2:** Effect of breed and ration on total DM intake and feed conversion ratio in Sokoto Gudali (SG) and Bunaji (BN) Nigerian bulls subjected to a short period (30-d) of feed restriction and 120 days of refeeding

Variables	Breed (B)		SEM	Diet (D)^1^		SEM	*P*-value	
	SG	BN		2% BW	1%BW		B	Diet
***DM intake, kg/d***								
Feeding restriction period, days 0–30								
Hay	3.07	3.04	0.02	2.14	3.96	0.02	0.09	< 0.0001
Total	6.92	6.96	0.06	9.48	4.41	0.08	0.99	< 0.0001
Early refeeding period, days 30–75								
Total	10.3	10.3	0.09	10.34	10.25	0.09	0.99	0.89
Final period to slaughter, days 75–134								
Total	10.2	10.2	0.09	10.12	10.33	0.09	0.99	0.20
Entire period, days 0–134								
Total	8.91	8.92	0.07	9.93	7.89	0.19	0.82	< 0.0001
***Feed conversion ration (DMI/ADG)***								
Feeding restriction period, days 0–30	7.44	6.99	0.58	6.51	7.92	0.56	0.45	0.02
Early refeeding period, days 30–75	6.92	7.42	0.62	8.72	5.63	0.61	0.44	< 0.0001
Final period to slaughter, days 75–134	6.29	5.78	0.34	6.45	5.61	0.33	0.14	0.01
Entire period, days 0–134	3.07	3.04	0.02	2.14	3.96	0.02	0.09	< 0.0001

Horizontal lines contain least square means for Nigerian cattle breeds and concentrate supplementation diets. Least squares means differ by Tukey’s test when *P* ≤ 0.05; SEM = standard error of the mean;

^1^Concentrate supplementation containing maize meal (60%), cotton cake (39%) and mineral mixture (1%)

Across the final period to slaughter (days 75 to 134), no significant breed effect (*P* > 0.05) on total DMI was observed. However, bulls receiving concentrate supplementation at 2% BW had higher total DM intake compared to animals receiving only 1% of BW ration (*P* < 0.0001). FCR was not affected by breed (*P* > 0.05) but was influenced by feed ration. Bulls on the restricted feed ration exhibited a higher FCR during the initial feeding phase (*P* < 0.05), while bulls on the high-concentrate diet showed a better FCR towards at the end of the study (*P* < 0.001). Overall, FCR was lower for bulls on the restricted diet (*P* < 0.05).

**Fig. 1:**
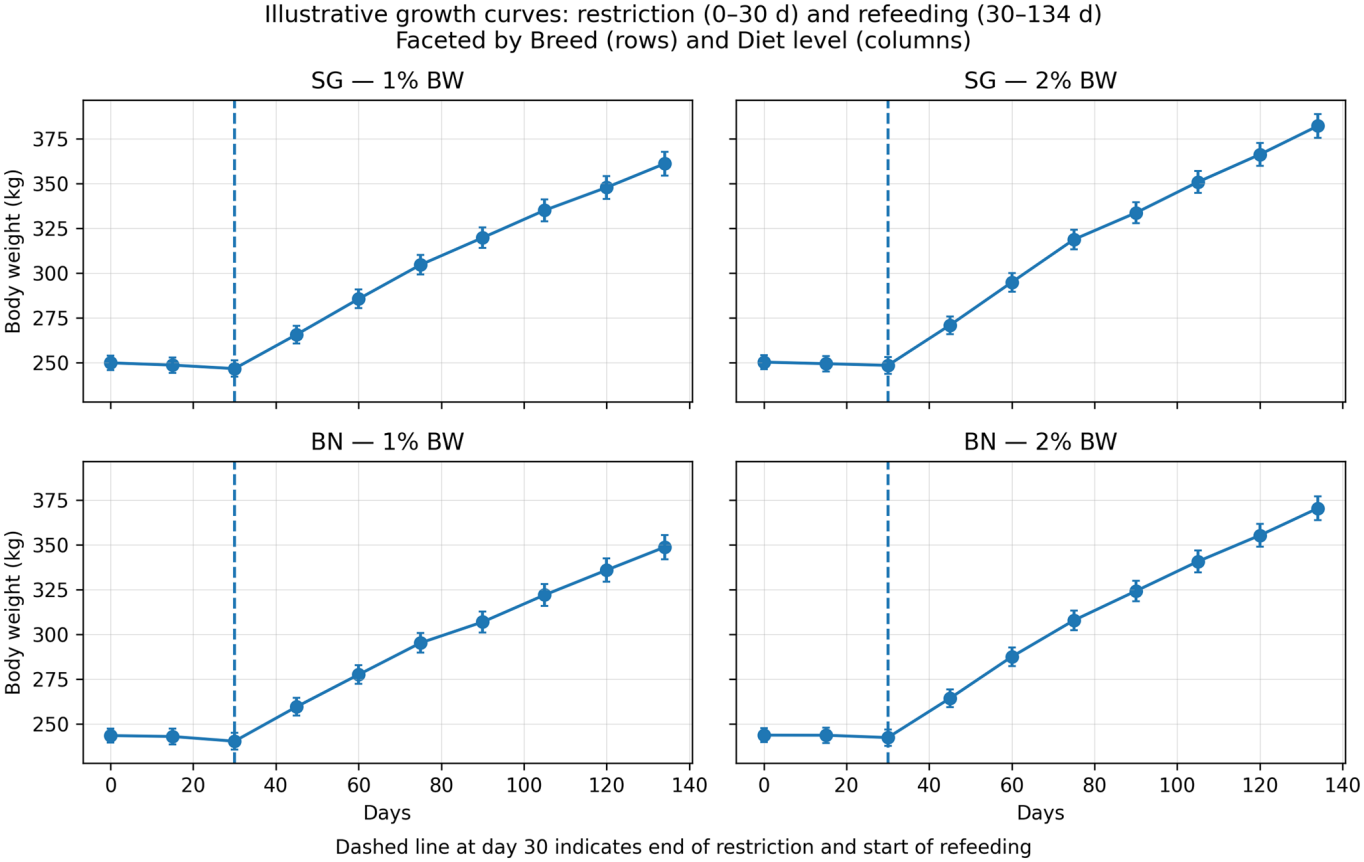
Growth curves of Nigerian bulls during feed restriction (days 0–30) and refeeding (days 30–134), faceted by breed (Sokoto Gudali and Bunaji) and dietary level (1 or 2% of body weight)

Initial live weights were similar between ration groups (*P* > 0.05), but bulls receiving concentrate supplementation at 2% BW were significantly (*P* < 0.001) heavier at the end of the trial (Table 3). Breed did not significantly affect live weight gain except during the refeeding mid-study period (days 75 to 117), where BN showed greater gains than SG Nigerian bulls (*P* = 0.001). Ration effects were pronounced during the initial 45 days (*P* < 0.001), with bulls receiving concentrate supplementation at 2% BW presented higher ADG than animals receiving 1% of BW concentrate diet (Fig. 1). However, during the latter half of the study, bulls receiving concentrate supplementation at 1% BW presented greater weight gains (*P* < 0.001). In addition, no significant differences (*P* > 0.05) on live weight gain were observed between dietary groups after day 75.

**Table 3:** Effect of breed and diet on live weight and average weight gain of Sokoto Gudali (SG) and Bunaji (BN) Nigerian bulls subjected to a short period (30-d) of feed restriction and 120 days of refeeding

Variables	Breed (B)		SEM	Diet (D)^1^		SEM	*P*-value	
	SG	BN		2% BW	1%BW		Breed	Diet
**Linear body measurements**								
Start, day-0	255	249	6.88	244	259	6.92	0.79	1.00
Feeding restriction period, days 0–30	288	278	7.08	292	260	7.06	0.99	< 0.001
Refeeding, day-75	296	286	6.75	304	280	6.89	0.99	< 0.001
Slaughter, day150	364	355	7.12	369	352	6.78	1.00	0.04
***Average weight gain (ADG)***,*** kg/d***								
Feeding restriction period, days 0–30	1.06	1.12	0.05	1.55	0.63	0.05	0.28	< 0.001
Refeeding period, days 30–75	1.50	1.50	0.09	1.26	1.74	0.09	0.98	< 0.001
Refeeding period, days 75–117	1.65	1.90	0.07	1.63	1.91	0.06	0.001	< 0.001
Refeeding period, days 117–134	1.34	1.33	0.09	1.34	1.33	0.09	0.89	0.87
Refeeding period, days 134–150	0.91	0.64	0.18	0.84	0.71	0.17	0.14	0.47
Entire period, days 0–150	1.25	1.26	0.04	1.33	1.18	0.04	0.81	< 0.004

Horizontal lines contain least square means for Nigerian cattle breeds and concentrate supplementation diets. Least squares means differ by Tukey’s test when *P* ≤ 0.05; SEM = standard error of the mean;

^1^Concentrate supplementation containing maize meal (60%), cotton cake (39%) and mineral mixture (1%)

### Linear measurements and body composition

No significant breed × feed ration interactions (*P* > 0.05) were found for linear measurements (Table 4). Breed influenced pelvic width, with SG Nigerian bulls showing greater values at the end of the feeding period (*P* < 0.05). Feed ration intake significantly affected all linear measurements (*P* < 0.05), with bulls receiving concentrate supplementation at 1% BW presenting greater measurements at the end of the study compared to animals fed high-concentrate diet (2% BW). Muscle depth was greater in BN bulls on days 75 and 150 (*P* < 0.05), while fat depth was higher in SG bulls from mid-study until the end of the trial (*P* < 0.05). Bulls on the high-concentrate diet had significantly greater muscle and fat depths during the first half of the study (*P* < 0.05), but no differences were noted during the final phase.

**Table 4:** Effect of breed and diet on body measurements, muscle and fat depth in Sokoto Gudali (SG) and Bunaji (BN) Nigerian bulls subjected to a short period (30-d) of feed restriction and 120 days of refeeding

Variables	Breed (B)		SEM	Diet (D)^1^		SEM	*P*-value	
	SG	BN		2% BW	1%BW		B	D
**Linear body measurements**								
**Height at withers**,** mm/kg**								
Start, day-0	3.69	3.86	0.10	3.73	3.82	0.10	0.76	0.99
Feeding restriction period, days 0–30	3.03	3.11	0.06	2.79	3.34	0.05	0.81	< 0.001
Refeeding, day-75	2.65	2.69	0.05	2.55	2.79	0.04	0.97	< 0.001
Slaughter, day-150	2.01	2.06	0.04	1.98	2.09	0.03	0.85	0.08
**Chest girth**,** mm/kg**								
Start, day-0	4.78	4.72	0.08	4.75	4.75	0.08	0.99	1.00
Feeding restriction period, days 0–30	4.34	4.39	0.06	4.13	4.59	0.05	0.99	< 0.001
Refeeding, day-75	3.91	3.90	0.05	3.79	4.02	0.05	1.00	0.001
Slaughter, day-150	3.29	3.24	0.10	3.26	3.27	0.10	0.99	1.00
**Length of back**,** mm/kg**								
Start, day-0	3.27	3.45	0.06	3.38	3.31	0.06	0.22	0.94
Feeding restriction period, days 0–30	2.71	2.72	0.04	2.50	2.93	0.04	1.00	< 0.001
Refeeding, day-75	2.27	2.33	0.04	2.23	2.37	0.04	0.94	0.03
Slaughter, day-150	1.81	1.88	0.05	1.78	1.90	0.04	0.75	0.21
**Chest depth**,** mm/kg**								
Start, day-0	1.85	1.93	0.04	1.87	1.90	0.03	0.47	0.99
Feeding restriction period, days 0–30	1.56	1.59	0.03	1.47	1.69	0.02	0.94	< 0.001
Refeeding, day-75	1.37	1.39	0.02	1.33	1.43	0.02	0.93	< 0.001
Slaughter, day-150	1.10	1.13	0.02	1.10	1.14	0.02	0.88	0.63
**Pelvic width**,** mm/kg**								
Start, day-0	1.29	1.44	0.08	1.43	1.29	0.07	0.63	0.71
Feeding restriction period, days 0–30	1.08	1.13	0.01	1.04	1.17	0.01	0.01	< 0.001
Refeeding, day-75	0.94	0.99	0.02	0.93	1.01	0.01	0.06	< 0.001
Slaughter, day-150	0.82	0.85	0.05	0.82	0.84	0.02	0.21	0.98
***Ultrasound measurements***								
**Muscle depth**,** mm**								
Start, day-0	44.2	46.7	1.37	45.9	45.01	1.31	0.78	0.99
Feeding restriction period, days 0–17	49.5	51.9	1.38	53.1	48.25	1.36	0.81	0.02
Feeding restriction period, days 17–30	50.9	54.2	1.39	56.0	49.05	1.36	0.38	< 0.001
Refeeding, day-75	55.6	60.4	1.40	59.0	57.04	1.37	0.03	0.90
Slaughter, day-150	58.7	67.1	1.41	62.5	63.33	1.39	< 0.001	0.99
**Fat Depth**,** mm**								
Start, day-0	0.79	0.61	0.06	0.74	0.67	0.06	0.15	0.97
Feeding restriction period, days 0–17	1.06	0.70	0.08	0.98	0.79	0.08	0.0013	0.48
Feeding restriction period, days 17–30	1.41	0.79	0.13	1.53	0.67	0.13	0.0004	< 0.001
Refeeding, day-75	3.51	2.51	0.28	3.42	2.60	0.28	0.021	0.12
Slaughter, day-150	7.41	5.14	0.48	6.77	5.78	0.48	0.001	0.55

Horizontal lines contain least square means for Nigerian cattle breeds and concentrate supplementation diets. Least squares means differ by Tukey’s test when *P* ≤ 0.05; SEM = standard error of the mean;

^1^Concentrate supplementation containing maize meal (60%), cotton cake (39%) and mineral mixture (1%)

### Carcass traits and non-carcass components

No significant breed × feed ration interactions (*P* > 0.05) were observed for any traits. Bunaji Nigerian bulls presented higher (*P* < 0.05) cold carcass weights, dressing percentages, and *M. longissimus thoracis* et lumborum area compared to SG bulls (Table 5). Conversely, SG bulls exhibited higher fat scores (*P* < 0.05). Ration treatment influenced carcass composition, with bulls on the high-concentrate ration showing higher cold carcass weights, perinephric-retroperitoneal fat, and rib joint fat composition (*P* < 0.05). Linear carcass measurements, including carcass length and depth, were greater in bulls receiving concentrate supplementation at 1% BW compared to bulls fed 2% of BW concentrate ration (*P* < 0.05).

**Table 5:** Effect of breed and diet on slaughter traits, 5-rib joint weight, *M. longissimus thoracis et lumborum* area, rib joint dissection and selected non-carcass components and carcass measurements of Sokoto Gudali (SG) and Bunaji (BN) Nigerian bulls subjected to a short period (30-d) of feed restriction and 120 days of refeeding

Variables	Breed (B)		SEM	Diet (D)^1^		SEM	*P*-value	
	SG	BN		2% BW	1%BW		Breed	Diet
Cold carcass weight, kg	192	201	5.9	202	192	5.5	0.02	0.0003
Dressing percentage, %	52.7	56.6	0.41	54.7	54.6	0.39	< 0.001	0.75
Carcass conformation	7.25	9.08	0.411	8.33	8.02	0.402	< 0.001	0.44
Fat covering	10.3	7.96	0.478	9.59	8.75	0.466	< 0.001	0.08
P-R^1^ fat, kg	8.76	8.56	0.798	9.34	7.99	0.778	0.72	0.03
P-R fat, g/kg	25.7	23.4	2.246	25.68	23.4	2.191	0.18	0.17
5-rib joint, kg	6.29	6.44	0.232	6.68	6.05	0.219	0.42	0.0006
*M. longissimus area*, cm/kg	0.18	0.21	0.012	0.19	0.20	0.012	0.004	0.29
***Rib joint composition***,*** g/kg***								
* M. longissimus*	170	207	10.86	184.14	192	10.590	< 0.001	0.31
Other muscle	208	213	14.20	203.87	218	13.610	0.66	0.18
Muscle trim	57.4	68.3	8.160	65.06	60.6	7.957	0.08	0.47
Bone and other tissue	178	177	5.811	174.46	180	5.535	0.92	0.16
Fat	139	86.9	13.07	124.94	101	12.740	< 0.001	0.02
Total muscle	237	265	11.9	245.90	259	11.570	< 0.001	0.06
***Non-carcass components***								
Heart, g/kg	3.20	3.04	0.167	3.02	3.21	0.163	0.21	0.13
Lungs, g/kg	8.61	8.26	0.551	8.33	8.54	0.529	0.40	0.61
Gall bladder, g/kg	0.86	0.87	0.088	0.86	0.87	0.088	0.75	0.74
Liver, g/kg	4.22	4.23	0.383	4.18	4.28	0.373	0.95	0.53
Spleen, g/kg	1.50	1.30	0.199	1.47	1.31	0.169	0.15	0.21
Intestines, g/kg	14.6	13.5	1.330	14.22	13.9	1.290	0.02	0.46
Rumen full, g/kg	46.7	43.5	2.627	32.50	47.1	2.468	0.14	0.05
Rumen empty, g/kg	13.9	13.3	0.637	13.07	14.2	0.622	0.23	0.03
Fore feet, g/kg	4.61	4.81	0.263	4.67	4.74	0.257	0.13	0.61
Hind feet, g/kg	4.89	4.97	0.234	4.84	5.02	0.222	0.48	0.13
Hide, g/kg	24.2	20.4	1.865	22.12	22.5	1.818	< 0.001	0.45
Kidney, g/kg	1.47	1.49	0.059	1.45	1.52	0.058	0.85	0.09
Head, g/kg	20.3	21.2	0.548	20.26	21.2	0.534	0.03	0.02
***Carcass measurements***								
Length of carcass, mm/kg	3.84	3.64	0.068	3.65	3.82	0.064	0.006	0.01
Carcass depth, mm/kg	1.39	1.32	0.030	1.31	1.40	0.029	0.04	0.004
Leg width, mm/kg	1.22	1.21	0.023	1.17	1.25	0.022	0.75	0.001
Leg thickness, mm/kg	0.83	0.82	0.014	0.80	0.84	0.014	0.49	0.005
Leg length, mm/kg	2.07	1.98	0.038	1.97	2.08	0.036	0.03	0.006

Horizontal lines contain least square means for Nigerian cattle breeds and concentrate supplementation diets. Least squares means differ by Tukey’s test when *P* ≤ 0.05; SEM = standard error of the mean;

^1^P-R is Perinephric-retroperitoneal;

^1^Concentrate supplementation containing maize meal (60%), cotton cake (39%) and mineral mixture (1%)

Non-carcass components such as intestinal, hide, and head weights differed significantly between breeds (*P* < 0.05). Sokoto Gudali bulls had heavier intestines and hides, whereas BN Nigerian bulls had heavier heads. Feed ration effects were also evident, with heavier rumen and kidney weights observed in bulls receiving concentrate supplementation at 1% BW (*P* < 0.05). Other non-carcass components were not significantly affected by breed or diet (*P* > 0.05).

## Discussion

Although SG bulls consumed more hay than BN bulls when receiving concentrate supplementation at 1% BW, no significant differences were observed between breeds under a high plane of nutrition. However, animals in the 2% BW concentrate supplementation group received a greater amount of concentrate to achieve higher overall consumption, potentially masking any breed effects.

According to Keogh et al. (2015a, 2015b); Silva et al. (2020), *ad libitum* feeding following a period of feed restriction in beef steers and bulls resulted in increased feed intake during the refeeding phase. However, the present study found no significant differences in dry matter intake (DMI) between rations during the refeeding period. This finding is consistent with Keogh et al. (2015a), who reported no variation in DMI during refeeding in Holstein Friesian bulls after a period of feed restriction, compared to their contemporaries maintained on a high plane of nutrition. Although feed intake did not differ between diets during the feeding restriction period, bulls receiving concentrate supplementation at 1% BW presented greater live weight gains compared to bulls receiving concentrate supplementation at 2% BW, leading to an improved feed conversion ratio (FCR).

Bulls supplemented at 1% concentrate as BW were more feed-efficient during refeeding than during the feeding restriction phase and outperformed bulls receiving concentrate supplementation at 2% BW throughout both phases (Fig. 1). Compensatory growth models have consistently demonstrated improvements in efficiency (Keogh et al. 2015a; Fitzsimons et al. 2017).

Several studies (Cuvelier et al. 2006; Albertí et al. 2008; Kostusiak et al. 2023) have indicated that late-maturing cattle breeds tend to have higher mature live weights and greater weight gains compared to early-maturing breeds. These traits are influenced by feeding intensity and age. In the present study, all bulls were purebred animals, with SG and BN cows naturally mated to Bunaji and Sokoto Gudali sires, respectively. Consequently, the expected breed differences in live weight were maintained, as evidenced by the significant weight differences between breeds throughout the trial. This result contrasts with the findings of Keane et al. (2011) and Kostusiak et al. (2024), who reported no breed effect on daily gains in crossbred Aberdeen Angus (AN) and Belgian Blue (BB) steers, as well as Holstein Friesian (HF) and HF × Charolais steers, respectively. Both SG and BN bulls exhibited similar responses to feed restriction and refeeding, with the only notable difference being in live weight gains midway through the refeeding period (days 60 to 65). Keady et al. (2021) examined the effects of dietary restriction and compensatory growth on performance, carcass characteristics, and metabolic hormone concentrations in AN and BB steers, but compensatory growth was not observed in AN steers during the finishing period.

The findings of the present study may be attributed to the lower initial weight of BN bulls at the onset of the trial. The duration of the feeding restriction phase was relatively short, aligning with Menegat et al. (2020), who stated that compensatory growth is enhanced when the restriction period is brief. In this study, bulls receiving concentrate supplementation at 1% BW presented higher compensatory growth early in the refeeding period compared to animals supplemented at 2% BW (Fig. 1). This finding is consistent with literature indicating that compensatory growth typically peaks between 30 and 60 days after the onset of refeeding and diet adaptation (Hornick et al. 2000). Although compensatory growth was observed in bulls fed 1% BW concentrate, it was insufficient to fully offset the potential growth lost during the feeding restriction phase.

This limited compensatory growth may be attributed to an insufficient level of feed restriction. Neel et al. (2007) reported that crossbred steers with a live weight gain of 0.23 kg/day during restriction exhibited greater compensatory growth during the finishing phase compared to steers gaining 0.45 and 0.68 kg/day during the restriction period. The present study yielded a compensatory growth (CG) recovery score of 45%, based on the formula CG Index (%) = [(ADG_restricted_​ − ADG_control_​) / ADG_control_​] × 100 (Ryan et al. 1993). Reported CG indices vary widely in the literature: Yambayamba et al. (1996) observed full (100%) recovery in heifers after 92 days of maintenance feeding, whereas Keogh et al. (2015a) found a 48% recovery following 55 days of refeeding after prolonged restriction. In general, CG indices range from 50% to 100% (Hornick et al. 2000), but outcomes depend on diet composition during restriction and refeeding, the severity and duration of restriction, and animal-related factors such as age, sex, and breed (Hornick et al. 2000).

In our study, breed differences in chest girth, back length, and chest depth were not detected, unlike the results of Campion et al. (2009a), who reported consistent divergence between beef breeds. As previously described in the literature (Alberti et al. 2008), narrower pelvic width is associated with delayed skeletal and muscular development. This pattern was initially evident in BN bulls, which displayed smaller pelvic width and reduced muscle depth early in the trial; however, these differences did not persist through slaughter, indicating effective skeletal compensation during refeeding.

By the end of the experiment, linear carcass measurements did not differ between feeding levels, consistent with Kamalzadeh et al. (1998), who showed that moderate feed restriction does not permanently constrain skeletal growth. The slightly greater linear dimensions observed in previously restricted bulls at slaughter are likely attributable to compensatory skeletal and muscular expansion during refeeding, a well-documented physiological response following nutrient limitation.

Regarding muscle traits, BN bulls maintained greater *M. longissimus thoracis* et *lumborum* depth throughout refeeding, reflecting their inherent muscularity (Hickey et al. 2007; Keane 2010). Although bulls fed 2% BW had higher muscle depth during the restriction period, animals fed 1% BW demonstrated clear compensatory gains, eliminating differences by slaughter. This agrees with Schoonmaker et al. (2004), who observed early divergences in muscle depth that disappeared after re-alimentation. Notably, the present study suggests that a compensatory growth-based feeding regimen does not negatively impact the economically significance of *M. longissimus thoracis et lumborum*.

## Conclusion

The short feed restriction period used in this study resulted in CG during the 120-day refeeding period, and had no effect on linear body measurements, ultrasonically scanned fat depth, carcass conformation, dressing percentage, and fat classification in SG and BN pure breed bulls. Although the bulls supplemented at 1% of their BW showed incomplete compensatory growth (index of 45%), the *M. longissimus thoracis* et *lumborum*, a tissue with great economic value, was able to recover completely, as evidenced by ultrasonically scanned muscle depth analysis.

## Data Availability

The data for this study is available on request from the corresponding author.
